# Disentangling Wording and Substantive Factors in the Spiritual Well-Being Scale

**DOI:** 10.1037/a0038001

**Published:** 2014-11-24

**Authors:** Aja L. Murray, Wendy Johnson, Alan J. Gow, Ian J. Deary

**Affiliations:** 1 Centre for Cognitive Ageing and Cognitive Epidemiology, Department of Psychology, University of Edinburgh; 2 Centre for Cognitive Ageing and Cognitive Epidemiology, Department of Psychology, University of Edinburgh, and Department of Psychology, Heriot-Watt University; 3 Centre for Cognitive Ageing and Cognitive Epidemiology, Department of Psychology, University of Edinburgh

**Keywords:** Spiritual Well-being Scale, dimensionality, method factors, religiosity

## Abstract

We evaluated the extent to which the Spiritual Well-Being Scale (SWBS) may help to meet the need for multidimensional, psychometrically sophisticated measures of spiritual and religious traits. Although the various forms of validity of the scale have, for the most part, been supported by psychometric studies, conflicting evidence surrounding its dimensionality has called into question its structural validity. Specifically, numerous authors have suggested that a more appropriate factor structure for the SWBS includes further substantive factors in addition to the 2 factors that the scale was originally intended to measure. In the current study, we attempted to resolve these debates using a combination of exploratory and confirmatory factor analysis based investigations in the Lothian Birth Cohort, 1921 study. Our analyses suggested that the additional factors suggested in previous studies may not have reflected substantive constructs; but rather, common variance due to methodological factors.

Religiosity is generally understood to be a multifaceted construct involving social, spiritual, and cognitive components. To optimize empirical research into religiosity, this multifaceted characterization should be reflected in the way in which the construct is operationalized in psychometric scales. We evaluated the Spiritual Well-Being Scale (SWBS; Paloutzian & Ellison, 1982)—a popular measure of religiosity which has been used in more than 300 published articles (Paloutzian, Bufford, & Widaman, 2012)—with respect to the number and nature of facets represented by the items of the scale.

Availability of psychometrically sophisticated measures of religiosity will be important in advancing study of the correlates of the construct. Previous research has yielded inconsistent relations among religiosity and its putative causes and consequences, leading to perception that these are both complex and poorly understood (Corsentino, Collins, Sachs-Ericsson, & Blazer, 2009; Zhang, 2010). One reason for these inconsistencies may be the use of suboptimal measures of religiosity which fail to capture and distinguish the many facets of religiosity. For example, evidence suggests that facets of religiosity show differential relations to mental and physical health outcomes (Corsentino et al., 2009). If these facets are not explicitly differentiated in psychometric measures, the result is likely to be a confused picture of how religiosity is related to these outcomes. Unfortunately, many studies use only a single item to measure religiosity. In doing so many facets of religiosity are conflated or omitted from examination entirely. Furthermore, single-item operationalizations of religiosity are liable to result in missing small associations with some criteria or outcomes because of attenuation of that association due to the unreliability of a single item (e.g., Mendoza & Mumford, 1987). By way of solution, we propose a renewed focus on operationalizing religiosity using multidimensional, psychometrically sophisticated measures with demonstrated utility.

We focused here on the SWBS as a potential multidimensional measure of religiosity because in addition to its popularity in empirical research, the scale has undergone extensive psychometric evaluation in a range of study populations (Utsey, Lee, Bolden, & Lanier, 2005). Previous psychometric studies have generally supported utility of the scale in various religious and nonreligious samples in terms of convergent validity, stability, and internal consistency (e.g., Bufford, Paloutzian, & Ellison, 1991; Ellison, 1983; Genia, 2001). However, the appropriate dimensionality of the scale remains contentious. Initially developed in conjunction with the social indicators movement, the SWBS was constructed to measure two aspects of spiritual well-being: religious well-being (RWB) and existential well-being (EWB). In its current form these constructs are measured by 10 items each. Scores on the RWB subscale are intended to reflect the degree to which individuals perceive that they have a positive and satisfying relationship with God. An example item is “My relationship with God helps me not to feel lonely.” Scores on the EWB scale are intended to reflect the extent to which individuals have a general sense of purpose and satisfaction with life. Thus, the EWB captures aspects of spiritual well-being which are not directly religious but which are more broadly spiritual. An example item is “I feel a sense of well-being about the direction my life is headed in.” The SWBS could, in principle, allow researchers to begin to unpack the associations between religious and spiritual traits and important outcomes because the scale separately identifies well-being associated with both religious beliefs directly and with spirituality in general.

However, several factor analytic studies of the scale have contributed evidence that the two factors suggested by the test developers are insufficient to describe the covariance among the 20 SWBS items (Utsey et al., 2005). These studies do not themselves agree on how many factors are necessary, what these factors are, or how they should be interpreted. Whereas some exploratory studies have apparently supported the two-factor structure originally proposed by the developers of the SWBS (Ellison, 1983; Genia, 2001), others have argued that three or more factors are more appropriate (e.g., Gow, Watson, Whiteman, & Deary, 2011; Miller, Fleming, & Brown-Anderson, 1998; Scott, Agresti, & Fitchett, 1998). Confirmatory factor analytic studies have also generally suggested that a two-factor structure does not represent good fit to the data across samples (Ledbetter, Smith, Fischer, & Vosler-Hunter, 1991; Utsey et al., 2005). A point of agreement across many of these studies is that the additional factors represent substantively meaningful constructs, rather than being due to some methodological artifact. For example, Scott, Agresti, and Fitchett (1998), labeled the three factors that they extracted as “Affiliation,” “Alienation,” and “Dissatisfaction with Life.” The “Affiliation” factor was interpreted as reflecting a sense of positive connection with God, and the “Alienation” factor as reflecting a sense of disconnection of self from God, and the “Dissatisfaction with Life” factor was interpreted as its name suggests.

We suggest that the conclusion that the additional factors identified when factor analyzing the SWBS are substantively meaningful may be inappropriate. Specifically, we suggest that the location of additional factors may be a result of two methodological artifacts: (a) method factors (also known as “nuisance factors” e.g., Millsap, 2011); and (b) combining samples of both religious and nonreligious respondents sampled from qualitatively distinct populations (Meredith & Teresi, 2006). If the hypothesis is correct, it implies that previous studies may have overextracted factors in the SWBS. Factor overextraction is undesirable because it can lead to the inclusion of superfluous constructs and a lack of model parsimony, as well as a degradation in the psychometric quality of subscales (Patil, Singh, Mishra, & Todd Donavan, 2008). However, underextraction of substantive factors can also have serious psychometric and substantive consequences because it can lead to important substantive constructs being missed (Patil et al., 2008). It is, therefore, important to consider carefully whether the additional factors identified in previous factor analyses of the SWBS can be considered to reflect theoretically and psychometrically useful constructs, or if they are best characterized as “nuisance factors” arising for methodological reasons.

Considering first the issue of method factors, Maydeu-Olivares and Coffman (2006) noted that a commonly observed type of nonsubstantive factor relates to item wording. Although a set of items may have been designed to measure the same construct, it is not uncommon for all the positively worded items to load on one factor and all the negatively worded items to load on another. Positively worded items present statements tapping strong expression of the construct directly and ask participants to rate the extent to which the construct applies to them. These are phrased in desirable terms, for example, in the SWBS: “I feel good about my future” is a positively worded item. Negatively worded items reflect the opposite ends of the construct and are phrased in undesirable terms, for example, in the SWBS: “I don’t enjoy much about life” is a negatively worded item. Thus, where a single substantive factor is hypothesized on theoretical grounds, two factors might arise in practice. Table 1 shows the range of suggested factor and principal components analysis solutions from published studies of the SWBS. The top rows of Table 1 show the item-to-factor allocations suggested by the test developers. Subsequent rows detail the item-to-factor allocations from further factor analytic studies. We list item groupings under the labels RWB and EWB based on the similarities of item content from the replication studies but these factors have often been given different labels.[Table-anchor tbl1]

The configural patterns (i.e., which items loaded on which factors/components) from Table 1 suggest that, although there is reasonable agreement on which items tend to cluster together across studies, there is some inconsistency regarding whether two or more factors are optimal for describing the general pattern of clustering. There are also some suggestions in these results that, in studies that have supported more than the two intended factors, the additional factors may have their origins in methodological rather than substantive constructs. Specifically, the items loading on the additional factors appear to depend on whether the items are positively or negatively worded.

Scott et al. (1998), for example, found that exploratory factor analysis in a sample of psychiatric inpatients suggested three correlated factors but the latter two factors represented a splitting of the items of the RWB into two factors, one comprising positively worded items, and the other comprising negatively worded items. A similar phenomenon was observed by Gow, Watson, Whiteman, and Deary (2011) when analyzing the data utilized in the current study. In principal components analysis, they found that the EWB scale split into two components: one defined by positively worded items and one defined by negatively worded items. Gow et al. (2011), however, did not observe this phenomenon when they analyzed the data using an exploratory Mokken procedure, which yielded only a two-subscale solution. This solution corresponded to reduced EWB and RWB factors, after excluding items which remained unselected by the exploratory Mokken procedure, indicating that they did not reflect the intended constructs well. However, it is relevant that it was primarily the negatively worded items from both EWB and RWB that remained unselected, further suggesting the possibility of method artifacts. This suggests that this factor splitting may have had more to do with patterns of responses to differently worded items than to a substantively meaningful split.

Another potential source of additional and nonsubstantive factors relates to the use of aggregated samples of both religious and nonreligious individuals. Note that, in the RWB construct, it is not the extent to which an individual is religious, but the extent to which they are religious in a positive or adaptive way that is measured. Its explicit focus on the valence of religiosity, that is, “positive religiosity” versus “negative religiosity” is interesting. On the one hand, it allows the personal impact of religion (positive vs. negative) on a religious individual to be ascertained and, therefore, helps to separate out the possible beneficial and detrimental effects of religion that have been discussed in the literature (e.g., Seybold & Hill, 2001). On the other hand, it complicates the interpretation of item responses across religious and nonreligious individuals because items will have different meanings to these individuals. For example, a typical item in the scale is “I believe that God loves me and cares for me.” A nonreligious person would be expected to select the *strongly disagree* response option because they do not believe in God; therefore, in their view, this nonexistent God could not possibly love and care for them (note that there is no *not applicable* or *unsure* type of middle response option). If this same response option was selected by a religious person, however, it may not indicate an absence of a belief in God, but the absence of a feeling of being loved and cared for by God, whom they believe exists. Therefore, responding in this way may be expected to have quite different implications for a religious and nonreligious person. In particular, it might predict more adverse outcomes for a religious person if it is indicative of dissatisfaction with their religion and associated negative feelings, than it would for a nonreligious person in whom religion is merely absent but not an active source of dissatisfaction.

Variability in the performance of items across groups of this type is referred to as differential item functioning (DIF). When there is DIF across two groups who are factor analyzed together, this can result in the appearance of additional factors in the aggregated sample (Meredith & Teresi, 2006). In addition, because individuals who differ in whether they are religious or not would, overall, be expected to score at quite different ends of the scale on RWB, it is possible that in an aggregated sample, additional “severity factors” would appear (Meredith & Teresi, 2006). Both Gow et al. (2011) and Scott et al. (1998) analyzed samples which included both religious and nonreligious participants (this was also likely true of the undergraduate sample used by Miller, Fleming, and Brown-Anderson (1998) but is not explicitly stated). Thus, this provides further reason to think that factor solutions with more than two *substantive* constructs may not be optimal.

In the present study we reanalyzed the data from the Lothian Birth Cohort, 1921 (LBC1921; as analyzed by Gow et al., 2011) to attempt to resolve some of the questions about the number of substantive constructs that are measured by the SWBS. Whereas the study by Gow et al. (2011) reported some preliminary analyses of the dimensionality of the SWBS, they did not consider the possibility that the additional constructs that emerged in their analyses were nonsubstantive nor that the items may have been functioning differently across the religious and nonreligious individuals in their aggregated sample. In this study we explicitly addressed these possibilities. We hypothesized that there are only two substantive constructs measured by the SWBS, corresponding to EWB and RWB, and that additional covariance due to item wording and aggregating religious and nonreligious groups can result in the appearance that there are additional substantive factors.

## Method

### Sample

We utilized data from the LBC1921, the same sample used by Gow et al. (2011). Briefly, the LBC1921 is a longitudinal cohort study investigating the causes and associates of individual differences in cognitive ageing in a relatively healthy cohort of community-dwelling individuals. Participants of LBC1921 were all born in 1921. Most had completed the Scottish Mental Survey cognitive ability test in 1932, which was administered in June 1932 to almost everyone in the Scottish population born in 1921 and attending school. The LBC1921 was recruited between 1999 and 2001, with an original *N* of 550. For a comprehensive description of the sample, recruitment and testing procedures, refer to Deary, Whiteman, Starr, Whalley, and Fox (2004) and Deary, Gow, Pattie, and Starr (2012). The SWBS was administered to LBC1921 participants around the time of the second wave of data collection between 2003 and 2005, when participants were of a mean age of 83.4 (*SD* = 0.5). Data on the SWBS were available for 371 participants (152 males and 219 females).

Of these 371 participants, 230 reported being current church members (information on church membership was not available for two participants). These individuals comprise our religious subsample. All participants were probably either of Christian faith or of no faith based on the homogeneous age and background of the cohort. Those of faith were probably either Church of Scotland Protestants or Roman Catholics.

### Statistical Procedure

#### Data screening

We first evaluated suitability of the data for our proposed analytic method by examining the distributional properties, missingness, and communalities of items.

#### Group comparisons

As preliminary tests for the existence of differences between the religious and nonreligious individuals, we compared EWB and RWB scale scores and their correlations in the two groups using independent samples *t* tests. Significant differences in means and correlations might indicate both sample heterogeneity due to true group differences on the constructs and/or test bias but either could affect factor structure. To gauge whether significant differences were likely to be of practical importance, we also examined their effect size.

#### Exploratory factor analysis (EFA)

An exploratory factor solution was estimated in order to observe where items loaded in an unconstrained model with the numbers of factors suggested by previously published factor analytic studies of the SWBS. Gow et al. (2011) already conducted a preliminary assessment of scale dimensionality using exploratory Mokken and principal components analysis in this sample, basing their factor retention decision for the latter analysis on the Kaiser criterion and the scree plot. The Kaiser criterion method is strongly discouraged because its performance in simulation studies has repeatedly been shown to be poor, with the method having a strong tendency toward overextraction (Velicer, Eaton, & Fava, 2000). The scree plot method has shown inconsistent performance in simulation studies and is recommended only as an adjunct to other methods. We, therefore, added to these, tests of dimensionality using parallel analysis (Horn, 1965) and Velicer’s minimum average partial (MAP; Velicer, 1976). These methods have shown superior accuracy in simulation studies (Velicer et al., 2000). However, factor retention criteria are blind to the meaning of factors, and any criterion may be prone to the extraction of nonsubstantive factors in the presence of substantial common variance due to method factors. We examined factor solutions from two-, three-, and four-factor models irrespective of the number factors suggested by these retention criteria because these are the numbers typically suggested by previous studies. We used principal axis factoring with minimum residuals (minres) estimation to estimate model parameters. We considered factor loadings to be substantive when they were .30 or greater. We examined the pattern of substantive loadings to assess whether they were consistent with the hypothesis that substantial item covariance due to method factors was present.

#### Confirmatory factor analysis

We further assessed the relative importance of item wording as a source of item covariance by fitting a confirmatory factor model in which each item loaded on two factors: one related to the proposed substantive content area and one related to the way in which the item was worded (positively vs. negatively). Thus, the model specified four latent factors in total: two substantive factors corresponding to EWB and RWB and two “method” factors corresponding to “positive wording” and “negative wording.” The method factors were specified as orthogonal to one another and to EWB and RWB; however, EWB and RWB were allowed to correlate. For comparison we also fit the two-factor structure proposed by the test developers. In this model, we also allowed EWB and RWB to correlate. These analyses allowed us to obtain estimates of the relative contributions of wording factors and the substantive constructs to the variance in items.

Based on the results from the first stage of model fitting, we attempted to refine the item set such that we could attain an appropriate measurement model for use in empirical analyses. We did this first by selecting items that had high loadings on the intended substantive construct and only low loadings on the wording factors. As a first attempt we selected only items that had at least a loading of .40 on the intended substantive construct and had less than 40% of their explained variance due to the relevant wording factors. Our goal in this was to reduce or remove the necessity of the wording factors. Further selections, if necessary, were then made on an ad hoc basis depending on the results of these selections. Thus, although we were using CFA, we were using it in an exploratory manner in order to attain an appropriate measurement model for use in empirical analyses but which would require further validation in independent data.

Models were estimated in Mplus 6.11 using maximum likelihood estimation (ML). This is considered appropriate for items with five or more response categories (Rhemtulla, Brosseau-Liard, & Savalei, 2012). As the SWBS has six response categories, ML was deemed appropriate. Item covariance coverage was high (>0.95), meaning that all pairs of variables had no more than 5% of cases missing. ML estimation is appropriate for dealing with this low level of missingness (Enders, 2010). In all cases, scaling and identification were achieved by fixing latent factor variances to 1.0. Models were judged to be good-fitting based on CFI and TLI values of >0.95, RMSEA values of <0.05, and SRMR values <.08 (Beauducel & Wittmann, 2005; Hu & Bentler, 1999).

In addition, Akaike’s Information Criterion (AIC; Akaike, 1987) and Bayesian Information Criterion (BIC; Raftery, 1995) were used to compare the appropriateness of the fitted models. AIC and BIC are useful for such model comparisons because they are designed to take account of the differences in model parsimony by penalizing models with high levels of complexity (Vrieze, 2012). BIC had larger parsimony penalties than AIC for the present analyses due to sample size. BIC differences of >10 have been taken to reflect differences in model fit that are substantively significant (Raftery, 1995).

We also addressed the possibility that including both religious and nonreligious individuals contributed in factor analyses of the SWBS affects factor structure, promoting appearance of additional, nonsubstantive factors. We did not consider the religious and nonreligious subsamples to be of a sufficient size to conduct multigroup analyses. For example, a multigroup confirmatory factor analysis of measurement invariance would require upward of approximately 150 participants in each group. Instead, we duplicated all our analyses in only those individuals who were judged to be religious (*n* = 230). We defined “religious” as being a current member of a church and “nonreligious” as not being a current member of a church (this included individuals who were past members of a church). We compared the parameter estimates from the EFA and CFA models from the religious subsample with those from the full sample to evaluate whether there were any differences suggestive of DIF.

## Results

### Data Screening

Descriptive statistics for the 20 items of the SWBS are presented in Table 2. Item responses were scored on a 6-point scale from 1 = *strongly disagree* to 6 = *strongly agree*. Nine items are negatively worded and were reverse-scored. This means that higher scores on all items indicated higher degrees of spiritual well-being.[Table-anchor tbl2]

In the whole sample, item means ranged from 3.43 to 4.82 (*M* = 3.97, *SD* = 0.42), suggesting minimal variability in item “difficulty.” The sample mean of 3.97 suggested that participants were generally scoring closer to the end of the scale representing higher spiritual well-being. Item coefficients of variation (ratios of standard deviations to means as percentages) ranged from 26% to 56% (mean 34%), suggesting variability in responding may have been somewhat limited. No item had skew in excess of an absolute magnitude of 0.43 or kurtosis in excess of an absolute magnitude of 0.89.

The mean for negatively worded items was 4.03 and the mean for positively worded items was 3.93. This difference was statistically significant based on a paired samples *t* test, *t*(370) = −2.15, *p* = .03; however, our sample size was large, and the significance reflected the rather small standard deviations. The difference would not likely be considered of much substantive importance.

Mean standard deviation for the negatively worded items was 1.37 and mean standard deviation for the positively worded items was 1.30. Mean skew for both the negatively worded items and the positively worded items was −0.24. Therefore, the two sets of items appeared to have roughly similar properties in the sample.

In the religious subsample item means ranged from 3.91 to 4.90 (*M* = 4.29, *SD* = 0.26), suggesting that this subsample was scoring higher on the SWBS than the nonreligious subsample as expected. Item coefficients of variation ranged from 21% to 32% (*M* = 28%); therefore, the smaller *SD* and coefficient of variation in this group suggested that selecting this subsample reduced the variability in responding further. No item had skew in excess of an absolute magnitude of 0.67 or kurtosis in excess of an absolute magnitude of 0.56.

### Group Comparisons

Scores on the RWB were considerably higher in the religious individuals (*M* = 41.6, *SD* = 9.7) than in the nonreligious individuals (*M* = 27.6, *SD* = 11.4) and the difference was statistically significant, *t*(243.5) = 11.8, *p* < .001, Cohen’s *d* = 1.33. Scores on the EWB scale were moderately higher in the religious individuals (*M* = 44.2, *SD* = 7.3) than in the nonreligious individuals (*M* = 41.4, *SD* = 6.5) and this difference was statistically significant, *t*(355) = 3.67, *p* < .001, Cohen’s *d* = 0.41. The correlation between the RWB and EWB scores in the nonreligious group was *r* = −.15, *p* = .08, and in the religious group the correlation was *r* = .59, *p* < .001). This difference was statistically significant (z = 7.42, *p* < .001). The mean and correlational differences across religious and nonreligious subgroups point to the existence of heterogeneity within the aggregated sample which could also potentially affect factor structure. We explore the issue in more detail below.

### Exploratory Factor Analysis

#### Full sample

Item communalities ranged from .25 to .81 with a mean of .57 (*SD* = .18), supporting the appropriateness of factor analysis. Parallel analysis using the principal axis extraction method (PA-PAF) suggested the extraction of four factors and parallel analysis using the principal components extraction method (PA-PCA) suggested the extraction of three factors. The MAP criterion reached a minimum of .02 and suggested three factors. Visual inspection of a scree plot also suggested three to four factors. Factor loadings from oblimin-rotated two-, three-, and four-factor solutions are reported in Table 3.[Table-anchor tbl3]

In the two-factor solution, the pattern of loadings largely corresponded to that proposed by the test developers, with Factor 1 representing RWB and Factor 2 representing EWB. The only exception was Item 2, which loaded over .3 only on Factor 1 instead of Factor 2. In addition, Item 20 cross-loaded on Factor 1. This cross-loading of item 20 was also found in Scott et al. (1998) and Genia (2001). The correlation between Factor 1 and Factor 2 was .30.

In the three-factor solution and four-factor solution, it was apparent that the wording of items was an important determinant of which items loaded on which factors. In both solutions, the first two factors corresponded similarly to RWB and EWB, and subsequent factors were defined by almost all negatively worded items or all positively worded items.

#### Religious subsample

In the religious subsample, retention criteria also suggested extraction of three to four factors. The two-, three-, and four-factor oblimin rotated solutions from this sample are provided in parentheses in Table 3. In general, the configural structure was very similar to that of the whole sample. However, for all solutions a number of small differences were evident. These differences appeared to depend on whether an item was positively or negatively worded. For example, in the two-factor solution negatively worded items had higher loadings and positively worded items had lower loadings on the EWB factor. Similarly, in the four-factor solution, the RWB factor loadings were attenuated for negatively worded items and increased for the positively worded items relative to the full sample. This is preliminary evidence for differential item functioning across the religious and nonreligious individuals (e.g., Millsap, 2011). The EWB construct generally had weaker substantive loadings and its items had greater tendencies to load more strongly on the method factors.

#### Confirmatory factor analyses

Fit statistics for Models 1 and 2 in both the full sample and the religious-only subsample are provided in Table 4 and factor loadings for Models 1 and 2 in Table 5. Model 1 included only the two putative substantive constructs of the SWBS. Model 2 included these substantive constructs plus a positive and a negative wording factor. Thus, in Model 2 each item was influenced by two factors: one substantive construct (either EWB or RWB) and one wording factor (either positive wording or negative wording). In Model 2, an out-of-range parameter estimate meant that it was necessary to constrain the residual variance of one item to be small and positive (0.1) in the full sample.[Table-anchor tbl4][Table-anchor tbl5]

None of the models provided good fit to the data (see Table 4); however, inclusion of the wording factors improved fit in both samples. The CFI, TLI, and RMSEA all indicated better fit and difference in fit between these two models based on AIC and BIC magnitudes was also substantial (whole sample ΔAIC = 555.24; ΔBIC = 480.83; subsample ΔAIC = 341.00; ΔBIC = 272.24). However, examining the statistical significance of factor loadings suggested that only the negative wording factor was supported: the positive wording factor had numerous nonsignificant or negative loadings. This greater support for the negative wording factor was likely due to the fact that the EWB construct is both weaker and includes more negatively worded items (five items vs. three from the RWB).

In Model 3, we attempted to address the poor fit of Model 1 by excluding poorly performing items. Items were excluded if: they had loadings of less than .40 on the substantive factors, had more than 40% of their variance explained by the relevant wording factor in either the whole sample or religious subsample, or showed marked differences in the importance of item wording across the two samples (potentially indicative of DIF). This led to the exclusion of Items 2, 4, 6, 12, 16, and 18. Only one of these items (Item 16) was in the set that failed to be selected into a Mokken scale by the exploratory item selection algorithm in Gow et al., (2011). This is likely due to the fact that the exploratory Mokken procedure used by the authors employs a hierarchical algorithm that seeks unidimensional scales beginning with the items which correlate best with an estimate of the first latent trait. As a result, the algorithm failed to select a large number of negatively worded items into the two sets of almost exclusively positively worded items which were assigned to Mokken scales at the beginning of the item selection procedure. Our approach differs from this in that it explicitly models multidimensionality in item responses and aims to exclude those items heavily influenced by nonsubstantive factors and less well influenced by substantive factors.

In both the full and religious-only subsample, fit was improved relative to the full item set but acceptable fit was not achieved, suggesting that our attempt to achieve a good measurement model for the SWBS by item exclusion was not successful. We did not attempt to make further amendments to the model as this risked capitalization on chance.

## Discussion

The purpose of this study was to attempt to resolve previous inconsistencies in the evidence for the factor structure of the SWBS. Although the SWBS was designed to measure two substantive factors, subsequent studies have generally identified more than two substantive factors, but have disagreed on the optimal number and nature of factors measured by the scale. Using a combination of exploratory and confirmatory factor analytic techniques, we identified some scale properties which may have contributed to these inconsistencies and which potentially undermine the validity of the scale. First, our analyses suggested that item wording unduly influenced responding and resulted in covariance additional to that due to the latent substantive factors. This may have led to the retention of more factors than the intended two substantive factors in previous studies. This was particularly true of negatively worded items to which responses may reflect a trait such as neuroticism or negative affectivity as much as the intended construct. Neither removing items to which this limitation applied in particular, nor modeling item wording factors allowed us to achieve an acceptable measurement model for the SWBS. Second, we found some preliminary evidence of differential item functioning across religious and nonreligious individuals. This is an undesirable property for a scale routinely administered to both religious and nonreligious individuals, often within the same sample.

Examining the items suggested that the problems with the scale may lie in their style of construction. For example, Item 5 “I believe that God is impersonal and not interested in my daily situations” could conceivably tap at least four aspects of an individual: Belief in God or not, belief in a personal God, belief that God is personally interested, belief that God is interested at the level of day-to-day happenings. Items with multiple components such as this can make it difficult for participants to respond sensibly and increase the likelihood that they will respond randomly (e.g., Brown & Maydeu-Olivares, 2010).

Item multidimensionality can also create violations of the assumption of local independence conditional on the latent substantive factors if they contribute to the covariance among items over and above their covariance due to the latent substantive factors. We discussed and tested the possibility that positive versus negative wording was one such influence and found some evidence that this was the case. However, the wording of items suggests that additional violations of local independence could arise even after accounting for covariance due to positive versus negative framing due to other similarities of wording or contextualization. For example, sets of items are contextualized in the same way. Three items refer to an individual’s future and in one previous study (Miller et al., 1998), these three items together with a fourth item referring to satisfaction with life all loaded together on one additional factor. Thus, the poor fit of the scale and the tendency for additional factors to be suggested in exploratory analyses may be partly explained by the presence of a high degree of item complexity increasing measurement error and creating violations of local dependence. When the latent substantive constructs are strong, these violations of local independence may matter less; however, the loadings on the EWB factor suggested that this factor at least was somewhat weak. This may partly explain why the negative wording factor was supported in our CFA investigations when a positive wording factor was not: The EWB scale contains more negatively worded items than the RWB scale (five vs. three).

Another way in which additional factors can arise is when subgroups with noninvariant factor structure or marked differences in item means are factor analyzed together. Our initial analyses using scale scores suggested significant group differences in scale means and correlations across religious and nonreligious individuals. Following up on this basic observation, our EFA and CFA analyses suggested that there may be some differential item functioning across religious and nonreligious respondents. This was seen in the differences in the relative loadings of some items on both wording and substantive factors. However, restricting analyses to the religious-only subgroup did not fully resolve this issue because it restricted the variance in the items considerably. This suggested either that within religious (or specifically Christian in this case) groups, there was limited variability in degree of religiosity or, alternatively, that the items of the SWBS are not optimal with regards to capturing whatever variability does exist. The latter hypothesis suggests that further psychometric work might yield items capable of capturing individual differences in extents of religiosity.

Although the specific differences observed in scale means and correlations between religious and nonreligious participants may not have been accurate if measurement was not invariant due to differential item functioning, their general pattern probably was. Not surprisingly, RWB scores were much higher in religious than nonreligious participants, but EWB scores were moderately higher too. This suggests that, at least within this sample’s cultural and historical milieu, religiosity may well have been associated with overall well-being, in a manner consistent with the questionnaire’s design. The much lower (and even negative, although not formally significantly so) correlation in the nonreligious participants calls this into question though, as the EWB scale is very closely related to general, overall well-being. The absent-to-negative correlation in the nonreligious participants suggests the possibility that when participants were secure and stable in not being religious, they might even have a tendency toward greater overall well-being. This calls into question the conceptual design of the SWBS questionnaire.

Overall, our analyses provided some evidence that additional factors often identified in factor analyses of the SWBS represent trivial common variance due to the wording of items. Our study did have some limitations, however, which may affect the generalizability of this result. Although our sample size was larger than many of the sample sizes in previous factor analytic studies of the SWBS, we did not have a large enough number of nonreligious individuals to justify multigroup analyses to formally assess for differential item functioning. We also had only a fallible measure of whether individuals were religious because being a current church member is not completely synonymous with being religious, although the two are likely to be highly correlated. Our sample was also from a birth cohort study and was, therefore, relatively homogeneous in background. Participants were all very likely to be either Christian or nonreligious, with few if any other religions represented. Together, these features of the sample may have restricted variance in the substantive constructs of interest. When this occurs, nonsubstantive influences of the kind discussed above such as wording factors can gain relatively more influence in determining item covariation (Murray & Johnson, 2014). Another drawback of the sample is that all were older adults. Although it would be desirable to have a scale that is applicable across all age groups, there are some items in the scale which could potentially show differential item functioning across age groups and this could mean that our results are less applicable to other age groups. Specifically, three items (Items 6, 10, and 14) refer to a person’s future: “I feel unsettled about my future,” “I feel a sense of well-being about the direction my life is headed in,” and “I feel good about my future.” For older adults who are nearer the ends of their lives, these items may carry an entirely different meaning than they do for younger adults. Such items may show particular differences between religious and nonreligious older adults too if a religious person looks forward to going to heaven at the end of life.

Consistent with the underlying population of older adults aged 83 and living in Scotland at approximately the same time, our sample was comprised of a larger proportion of females than males (a male:female ratio of 0.62 in the current sample compared with a ratio of 0.69 in the population; National Records of Scotland, 2011). Therefore, it is possible that our results were influenced by the gender imbalance of the sample if the scale exhibits differential scale functioning by gender. To our knowledge, no previous studies have investigated differential functioning of the SWBS by gender, making this a potentially important future research direction.

The possibility that the scale functions differently across age, gender, or religious groups highlights that scale scores are not inherently “reliable” or “valid” but have psychometric properties conditional on the particular population from which participants are sampled. Thus, scale performance needs to be evaluated in samples spanning the entire range of participants for whom its use is intended as well as assessed for differential item functioning across key subgroups. Sass (2011) noted that differential item functioning testing should play a key role in item selection at the test development stage and it may be even more important to select items that are free from differential item functioning than to select those that have high factor loadings.

Finally, we employed CFA but did so in an exploratory manner. That is, we used CFA to identify the sources of nonsubstantive covariance among items, rather than as a confirmatory test of a specific hypothesized structure. Therefore, our CFA should not be considered confirmatory in the usual sense.

The validity issues identified in the current study can be used to inform the empirical application and revision of the SWBS or the development of new measures to measure spiritual and religious traits. We offer the following suggestions for developing new measures of religiosity or revising the SWBS:

1 **Content specification of substantive factors.** The substantive factors that the scale aims to measure should be carefully defined and close attention paid to whether items match this specification, rather than reflecting unintended constructs.
2 **Item difficulty.** Write items that tap a wider range of the religious and spiritual traits so that the scale can accurately measure individuals who are both low and high on the traits.
3 **Item wording.** Simplify the wording of items, avoiding multiple clauses or qualifiers.
4 **Differential item functioning.** Item performance across religious and nonreligious individuals as well as across individuals of different religions should be evaluated when assessing items for inclusion in a religiosity scale.

In addition, we offer the following recommendations with respect to using the SWBS in future empirical studies:

1 **Scoring.** In empirical analyses, scoring the SWBS based on the two-factor structure intended by the test developers may be more appropriate than scoring the scale based on three- or four-factor structures which have been identified in subsequent factor analytic studies. Using more than two factors risks degrading the reliability of the scales; however, the disadvantage of this approach is that it conflates variance due to wording and variance due to the substantive constructs. An alternative would be to use latent or factor scores from a measurement model similar to that used in the current study.
2 **Factor analyses.** The possibility that additional factors identified when factor analyzing religiosity scales are nonsubstantive should be considered to protect against overfactoring.

## Conclusion

We attempted to resolve previous debates surrounding the factor structure of the SWBS. We identified several features of the SWBS that may have contributed to disagreements on the nature and number of the substantive factors that it measures: differential item functioning across religious and nonreligious individuals, additional item covariance due to item wording, and item complexity. Excluding nonreligious individuals, excluding poorly performing items or modeling covariance due to item wording did not lead to an acceptable measurement model. Although, collectively, this calls into question the structural validity of the scale, the issues identified can inform the revision of the scale or the development of new scales to measure spiritual and religious traits. It also suggests that future application of the SWBS in empirical studies may benefit from focusing on the two substantive factors that the scale was originally developed to assess, rather than specifying additional factors which may simply reflect the presence of common variance due to methodological artifacts.

## Figures and Tables

**Table 1 tbl1:** Configural Structures of Spiritual-Wellbeing Scale (SWBS) From Previous Exploratory Factor Analytic Studies

Factor label	Proposed factors (Ellison, 1983)					
	RWB	EWB				
Items	**1**, 3, **5**, 7, **9**, 11, **13**, 15, 17, 19	**2**, 4, **6**, 8, 10, **12**, 14, **16**, **18**, 20				
Replication study			Additional Factor^a^ 1	Additional Factor 2	Additional Factor 3	Removed items
Gow et al. (2011) PCA	**1**, 2, 3, **5**, 7, **9**, 11, **13**, 15, 17, 19	4, 8, 10, 14, 20	**2, 6, 12, 16, 18**			
Gow et al. (2011) Mokken	3, 7, 11, 15, 17, 19	4, 8, 10, **12**, 14, **18**				**1, 2, 5**, **6, 9, 13**, **16**, 20
Scott et al. (1998)	3, 7, 11, 15, 17, 19, 20	**6, 12, 16**	**1**, **2, 5, 9**, **13, 18**			4, 8, 10, 14
Genia (2001) Oblimin solution	**1**, 3, **5**, 7, **9**, 11, **13**, 15, 17, 19, 20	**2**, 4, **6**, 8, 10, 11, **12**, 14, **16, 18**, 20				
Miller et al. (1998) Caucasian sample	**1**, 3, **5**, 7, **9**, 11, **13**, 15, 17, 19	4, 8, **12**, **16, 18,** 20	**2**, **6**, **10**, 14			
Miller et al. (1998) African-American sample	11, 15, 17, 19	**6,** 8, 10, 14	**5, 9, 12**, **13, 16, 18**	**1**, 3, 4	**2**, 7, 20	
*Note.* Negatively worded items are shown in boldface. PCA = principal components analysis.						
^a^ We use the term “factor” in a generic sense to mean both “factor” (for exploratory Mokken and factor analyses) and “component” (for principal component analyses).						

**Table 2 tbl2:** Descriptive Statistics for Spiritual Well-Being Scale (SWBS) Items in LBC1921 Data: Whole Sample (Religious Only Subsample in Parentheses)

Item no.	*N*	Mean	*SD*	Skew	Kurtosis
**1**	**366 (228)**	**3.62 (4.10)**	**1.56 (1.31)**	**−0.09 (−0.08)**	**−0.79 (−0.56)**
**2**	**368 (228)**	**4.01 (4.26)**	**1.41 (1.26)**	**−0.28 (−0.25)**	**−0.40 (−0.19)**
3	367 (229)	3.93 (4.52)	1.55 (1.18)	−0.43 (−0.40)	−0.51 (0.28)
4	367 (227)	4.59 (4.69)	1.03 (0.99)	−0.17 (0.01)	0.65 (−0.07)
**5**	**362 (225)**	**3.85 (4.33)**	**1.50 (1.27)**	**−0.23 (−0.27)**	**−0.55 (−0.22)**
**6**	**367 (227)**	**4.11 (4.14)**	**1.28 (1.21)**	**−0.11 (−0.06)**	**−0.38 (−0.33)**
7	359 (224)	3.39 (3.91)	1.49 (1.27)	−0.06 (−0.19)	−0.73 (−0.24)
8	367 (227)	4.23 (4.32)	1.11 (1.09)	−0.22 (−0.18)	−0.05 (−0.09)
**9**	**362 (225)**	**3.70 (4.16)**	**1.49 (1.29)**	**−0.15 (−0.24)**	**−0.63 (−0.27)**
10	366 (227)	4.16 (4.24)	1.06 (1.01)	−0.21 (−0.13)	0.50 (0.54)
11	365 (227)	3.62 (4.18)	1.47 (1.15)	−0.25 (−0.13)	−0.50 (0.18)
**12**	**368 (228)**	**4.82 (4.90)**	**1.08 (1.15)**	**−0.71 (−0.67)**	**−0.03 (−0.07)**
**13**	**361 (225)**	**3.49 (3.99)**	**1.44 (1.26)**	**−0.07 (−0.14)**	**−0.54 (−0.18)**
14	363 (225)	4.22 (4.34)	1.26 (1.07)	−0.28 (−0.28)	0.55 (0.25)
15	363 (225)	3.63 (4.20)	1.44 (1.21)	−0.21 (−0.31)	−0.48 (0.07)
**16**	**364 (226)**	**4.08 (4.15)**	**1.26 (1.17)**	**−0.25 (−0.19)**	**−0.17 (0.08)**
17	364 (227)	3.43 (3.96)	1.43 (1.21)	−0.11 (−0.10)	−0.54 (−0.15)
**18**	**364 (226)**	**4.54 (4.63)**	**1.18 (1.16)**	**−0.27 (−0.32)**	**−0.27 (−0.26)**
19	360 (224)	3.64 (4.19)	1.47 (1.17)	−0.26 (0.12)	−0.49 (0.08)
20	362 (225)	4.35 (4.52)	1.12 (1.01)	−0.40 (0.00)	0.89 (0.07)
*Note.* Negatively worded items are shown in boldface. RWB items are odd-numbered and EWB items are even-numbered.					

**Table 3 tbl3:** Oblimin-Rotated Exploratory Factor Solutions in LBC1921 Data: Whole Sample (Religious Only Subsample in Parentheses)

	Two-Factor solution		Three-Factor solution			Four-Factor solution			
Item no.	Factor 1 (RWB)	Factor 2 (EWB)	Factor 1 (RWB)	Factor 2 (EWB)	Factor 3 (Negative)	Factor 1 (RWB)	Factor 2 (EWB)	Factor 3 (Positive)	Factor 4 (Negative)
**1**	**.67 (.46)**		**.65 (.57)**		**.35 (.35)**	**.81 (.49)**			**(.45)**
**2**	**.40 (.48)**		**.38 (.52)**		**.35**	**.55 (.38)**			**(.32)**
3	.82 (.76)		.82 (.72)					.92 (.82)	
4		.47		.51 (.40)			.43	.37 (.62)	
**5**	**.72 (.57)**		**.69 (.64)**	**.36**	**(.32)**	**.63 (.32)**		**(.36)**	**(.37)**
**6**		**.55 (.59)**			**.49 (.52)**		**.39**		**.37 (.57)**
7	.85 (.86)		.86 (.82)			.50 (.82)			
8		.70 (.38)		.69 (.55)			.73 (.56)		
**9**	**.74 (.32)**		**.70 (.65)**		**.36 (.32)**	**.75 (.36)**			**(.38)**
10	(.37)	.72 (.38)		.75 (.66)			.79 (.62)		
11	.86 (.56)		.86 (.77)			.33 (.48)		.59 (.43)	
**12**		**.53 (.77)**		**.33**	**.52 (.69)**				**.58 (.67)**
**13**	**.78 (.70)**		**.76 (.83)**			**.84 (.67)**			**(.32)**
14		.74 (.44)		.74 (.71)			.75 (.63)		
15	.86 (.83)		.88 (.75)			.40 (.62)		.45	
**16**		**.42 (.66)**			**.53 (.65)**				**.46 (.67)**
17	.91 (.92)		.92 (.87)			.69 (.81)			−.36
**18**		**.53 (.77)**		**.32**	**.58 (.72)**				**.57 (.74)**
19	.86 (.89)		.87 (.82)			.54 (.82)			
20	.32 (.57)	.46	.36 (.41)	.47 (.47)			.50 (.41)		
*Note.* Negatively worded items are shown in boldface. Not showing loadings < |.30|. RWB items are odd-numbered and EWB items are even-numbered.									

**Table 4 tbl4:** Fit Statistics for Confirmatory Models of SWBS in LBC1921 Data

Model	χ^**2**^	*df*	CFI	TLI	RMSEA	SRMR	AIC	BIC
Model 1: Substantive factors	1060.32	169	.81	.78	.12	.11	21051.98	21290.87
Model 2: Substantive factors plus wording factors	467.08	150	.93	.91	.08	.09	20496.74	20810.04
Model 3: Substantive factors with reduced item set	528.721	91	.88	.86	.13	.08	14227.96	14396.24
Model 1 in religious-only sample	796.04	169	.77	.75	.13	.10	12197.09	12406.81
Model 2 in religious-only sample	415.04	149	.90	.88	.09	.09	11856.09	12134.57
Model 3 in religious-only sample	341.40	91	.87	.85	.12	.11	8211.00	8222.55

**Table 5 tbl5:** Factor Loadings on Existential Well-Being (EWB), Religious Well-Being (RWB), and Wording Factors in Model 2 Fit to LBC1921 Data: Whole Sample (Religious Only Subsample in Parentheses)

Item no.	EWB	RWB	Positive wording	Negative wording	Proportion of total item variance explained	Proportion of total item variance explained by wording	Proportion of explained item variance due to wording
**1**		**.66 (.55)**		**.45 (.43)**	**.63 (.48)**	**.20 (.18)**	**.31 (.38)**
**2**^b^	**.29 (.44)**			**.37 (.25)**	**.22 (.26)**	**.14 (.06)**	**.64 (.23)**
3^a^		.79 (.82)	−.57 (.38)		— (.97)	— (.14)	— (.14)
4^b^	.49 (.54)		−.23 (.54)		.29 (.43)	.05 (.29)	.17 (.67)
**5**		**.70 (.67)**		**.39 (.40)**	**.64 (.63)**	**.15 (.16)**	**.23 (.25)**
**6**^b^	**.48 (.50)**			**.39 (.41)**	**.38 (.41)**	**.15 (.17)**	**.39 (.41)**
7		.85 (.80)	−.02 (−.21)		.72 (.68)	.00 (.04)	.00 (.06)
8	.73 (.69)		.06 (−.07)		.53 (.49)	.00 (.00)	.00 (.00)
**9**		**.70 (.66)**		**.48 (.41)**	**.72 (.60)**	**.23 (.17)**	**.32 (.28)**
10	.78 (.78)		.07 (−.05)		.62 (.61)	.00 (.00)	.00 (.00)
11		.84 (.82)	−.25 (.11)		.77 (.68)	.06 (.01)	.08 (.01)
**12**^b^	**.46 (.47)**			**.44 (.53)**	**.41 (.50)**	**.19 (.28)**	**.46 (.56)**
**13**		**.75 (.74)**		**.41 (.34)**	**.73 (.66)**	**.17 (.12)**	.23 (.18)
14	.76 (.77)		.03 (−.02)		.58 (.59)	.00 (.00)	.00 (.00)
15		.87 (.84)	−.15 (−.02)		.79 (.71)	.02 (.00)	.03 (.00)
**16**^b^	**.35 (.34)**			**.45 (.57)**	**.32 (.63)**	**.20 (.32)**	**.63 (.51)**
17		.93 (.87)	.10 (−.14)		**.88 (.79)**	.01 (.02)	.01 (.02)
**18**^b^	**.51 (.50)**			**.51 (.62)**	**.52 (.63)**	**.26 (.38)**	**.50 (.60)**
19		.87 (.87)	.01 (−.17)		**.76 (.79)**	.00 (.03)	.00 (.04)
20	.57 (.71)		−.05 (−.02)		.33 (.51)	.00 (.00)	.00 (.00)
*Note.* Negatively worded items are shown in boldface. RWB items are odd-numbered and EWB items are even-numbered.							
^a^ The residual variance of this item was constrained to be small and positive due to an improper solution. The parameter estimates reported are from Model 2 estimated in the full sample and religious only subsample (in parentheses). ^b^ Denotes an item which had a loading on the relevant substantive factor of less than .40 or had more than 40% of its explained variance due to the relevant wording factor.							

## References

[c1] Akaike H. (1987). Factor analysis and AIC. Psychometrika, 52, 317–332. doi:10.1007/BF02294359

[c2] Beauducel A., & Wittmann W. W. (2005). Simulation study on fit indexes in CFA with slightly distorted simple structure. Structural Equation Modeling, 12, 41–75. doi:10.1207/s15328007sem1201_3

[c3] Brown A., & Maydeu-Olivares A. (2010). Issues that should not be overlooked in the dominance versus ideal point controversy. Industrial and Organizational Psychology, 3, 489–493. doi:10.1111/j.1754-9434.2010.01277.x

[c4] Bufford R. K., Paloutzian R. F., & Ellison C. W. (1991). Norms for the spiritual well-being scale. Journal of Psychology and Theology, 19, 56–70.

[c5] Corsentino E. A., Collins N., Sachs-Ericsson N., & Blazer D. G. (2009). Religious attendance reduces cognitive decline among older women with high levels of depressive symptoms. The Journals of Gerontology Series A: Biological Sciences and Medical Sciences, 64A, 1283–1289. doi:10.1093/gerona/glp116 PMC2773810 19675176

[c6] Deary I. J., Gow A. J., Pattie A., & Starr J. M. (2012). Cohort profile: The Lothian Birth Cohorts of 1921 and 1936. International Journal of Epidemiology, 41, 1576–1584. doi:10.1093/ije/dyr197 22253310

[c7] Deary I. J., Whiteman M. C., Starr J. M., Whalley L. J., & Fox H. C. (2004). The impact of childhood intelligence on later life: Following up the Scottish Mental Survey of 1932 and 1947. Journal of Personality and Social Psychology, 86, 130–147. doi:10.1037/0022-3514.86.1.130 14717632

[c8] Ellison C. W. (1983). Spiritual well-being: Conceptualization and measurement. Journal of Psychology and Theology, 11, 330–340.

[c9] Enders C. K. (2010). Applied missing data analysis. New York, NY: Guilford Press.

[c10] Genia V. (2001). Evaluation of the Spiritual Well-Being Scale in a sample of college students. The International Journal for the Psychology of Religion, 11, 25–33. doi:10.1207/S15327582IJPR1101_03

[c11] Gow A. J., Watson R., Whiteman M., & Deary I. J. (2011). A stairway to heaven? Structure of the religious involvement inventory and spiritual well-being scale. Journal of Religion and Health, 50, 5–19. doi:10.1007/s10943-010-9375-2 20614185

[c12] Horn J. L. (1965). A rationale and test for the number of factors in factor analysis. Psychometrika, 30, 179–185. doi:10.1007/BF02289447 14306381

[c13] Hu L. T., & Bentler P. M. (1999). Cut-off criteria for fit indexes in covariance structure analysis: Conventional criteria versus new alternatives. Structural Equation Modeling, 6, 1–55. doi:10.1080/10705519909540118

[c14] Ledbetter M. F., Smith L. A., Fischer J. D., & Vosler-Hunter W. L. (1991). An evaluation of the construct validity of the Spiritual Well-Being Scale: A confirmatory factor analytic approach. Journal of Psychology and Theology, 19, 94–102.

[c15] Maydeu-Olivares A., & Coffman D. L. (2006). Random intercept item factor analysis. Psychological Methods, 11, 344–362. doi:10.1037/1082-989X.11.4.344 17154751

[c16] Mendoza J. L., & Mumford M. (1987). Corrections for attenuation and range restriction on the predictor. Journal of Educational and Behavioral Statistics, 12, 282–293. doi:10.3102/10769986012003282

[c17] Meredith W., & Teresi J. A. (2006). An essay on measurement and factorial invariance. Medical Care, 44, S69–S77. doi:10.1097/01.mlr.0000245438.73837.89 17060838

[c18] Miller G., Fleming W., & Brown-Anderson F. (1998). Spiritual Well-Being Scale ethnic differences between Caucasians and African Americans. Journal of Psychology and Theology, 26, 358–364.

[c19] Millsap R. E. (2011). Statistical approaches to measurement invariance. New York, NY: Routledge.

[c20] Murray A., & Johnson W. (2014). The effect of sample truncation on the number of factors to retain in exploratory factor analysis. Manuscript submitted for publication.

[c21] National Records of Scotland (2011). Scotland census release 1C: Statistical bulletin. Retrieved from http://www.scotlandscensus.gov.uk/release-1-statistical-bulletin

[c22] Paloutzian R. F., Bufford R. K., & Widaman A. J. (2012). Spiritual Well-Being Scale: Mental and physical health relationships In Cobb M., Puchalski C. M., & Rumbold B. (Eds.), Oxford textbook of spirituality in healthcare (pp. 353–358). New York, NY: Oxford University Press. doi:10.1093/med/9780199571390.003.0048

[c23] Paloutzian R. F., & Ellison C. W. (1982). Loneliness, spiritual well-being and the quality of life In Peplau L. A. & Perlman D. (Eds.), Loneliness: A sourcebook of current theory, research and therapy (pp. 224–237). New York, NY: Wiley.

[c24] Patil V. H., Singh S. N., Mishra S., & Todd Donavan D. (2008). Efficient theory development and factor retention criteria: Abandon the “eigenvalue greater than one” criterion. Journal of Business Research, 61, 162–170. doi:10.1016/j.jbusres.2007.05.008

[c25] Raftery A. E. (1995). Bayesian model selection in social research. Sociological Methodology, 25, 111–163. doi:10.2307/271063

[c26] Rhemtulla M., Brosseau-Liard P. E., & Savalei V. (2012). When can categorical variables be treated as continuous? A comparison of robust continuous and categorical SEM estimation methods under suboptimal conditions. Psychological Methods, 17, 354–373. doi:10.1037/a0029315 22799625

[c27] Sass D. A. (2011). Testing measurement invariance and comparing latent factor means within a confirmatory factor analysis framework. Journal of Psychoeducational Assessment, 29, 347–363. doi:10.1177/0734282911406661

[c28] Scott E. L., Agresti A. A., & Fitchett G. (1998). Factor analysis of the “Spiritual Well-Being Scale” and its clinical utility with psychiatric inpatients. Journal for the Scientific Study of Religion, 37, 314–321. doi:10.2307/1387530

[c29] Seybold K. S., & Hill P. C. (2001). The role of religion and spirituality in mental and physical health. Current Directions in Psychological Science, 10, 21–24. doi:10.1111/1467-8721.00106

[c30] Utsey S. O., Lee A., Bolden M. A., & Lanier Y. (2005). A confirmatory test of the factor validity of scores on the Spiritual Well-Being Scale in a community sample of African Americans. Journal of Psychology and Theology, 33, 251–257.

[c31] Velicer W. F. (1976). Determining the number of components from the matrix of partial correlations. Psychometrika, 41, 321–327. doi:10.1007/BF02293557

[c32] Velicer W. F., Eaton C. A., & Fava J. L. (2000). Construct explication through factor or component analysis: A review and evaluation of alternative procedures for determining the number of factors or components In Goffin R. D. & Helmes E. (Eds.), Problems and solutions in human assessment: Honoring Douglas N. Jackson at seventy (pp. 41–71). Boston, MA: Kluwer. doi:10.1007/978-1-4615-4397-8_3

[c33] Vrieze S. I. (2012). Model selection and psychological theory: A discussion of the differences between the Akaike Information Criterion (AIC) and the Bayesian Information Criterion (BIC). Psychological Methods, 17, 228–243. doi:10.1037/a0027127 22309957 PMC3366160

[c34] Zhang W. (2010). Religious participation, gender differences, and cognitive impairment among the oldest-old in China. Journal of Aging Research, 2010, 1–10. doi:10.4061/2010/160294 PMC2990098 21152194

